# Rhizospheric bacteria of maize with potential for biocontrol of *Fusarium verticillioides*

**DOI:** 10.1186/s40064-016-1780-x

**Published:** 2016-03-15

**Authors:** Alejandro Miguel Figueroa-López, Jesús Damián Cordero-Ramírez, Juan Carlos Martínez-Álvarez, Melina López-Meyer, Glenda Judith Lizárraga-Sánchez, Rubén Félix-Gastélum, Claudia Castro-Martínez, Ignacio Eduardo Maldonado-Mendoza

**Affiliations:** Centro Interdisciplinario de Investigación para el Desarrollo Integral Regional Unidad Sinaloa (CIIDIR-Unidad Sinaloa), Instituto Politécnico Nacional, Blvd. Juan de Dios Bátiz Paredes No. 250, AP 280. Col. San Joachin, CP 81101 Guasave, Sinaloa Mexico; Unidad Los Mochis, Depto. de Ciencias Biológicas, Universidad de Occidente, Blvd. Macario Gaxiola y Carr. Internacional s/n, CP 81223 Los Mochis, Sinaloa Mexico

**Keywords:** *Fusarium verticillioides*, Antagonists, PGPR, Biocontrol microorganisms

## Abstract

The stalk, ear and root rot (SERR) of maize caused by *Fusarium verticillioides* (*Fv*) severely impacts crop production in tropical and subtropical regions. The aim of the present work was to screen bacterial isolates in order to find novel native biocontrol agents against *Fv*. A culturable bacterial collection consisting of 11,520 isolates enriched in Firmicutes and Proteobacteria was created from rhizosphere samples taken from SERR symptomatic or asymptomatic maize plants. The complete collection was screened for potential activity against *Fv* using a liquid antagonism assay followed by dual cultures in solid medium, selecting for 42 bacteria (*Bacillus*, *Pseudomonas* and *Paenibacillus*) that inhibit *Fv* growth (>45 %). *In planta* assays demonstrated that three *Bacillus* isolates: *B*. *megaterium* (*B*5), *B*. *cereus* sensu lato (*B*25) and *Bacillus* sp. (*B*35) displayed the highest antagonistic activity against *Fv*. Pot experiments performed in a greenhouse with *Bacillus cereus* sensu lato B25 confirmed these findings and showed a reduction of *Fv* disease severity and incidence on plants. Antagonistic activity analysis revealed that these strains produce glucanases, proteases or chitinases, as well as siderophores and auxins and suggests these as possible control mechanisms against *Fv*.

**Electronic supplementary material:**

The online version of this article (doi:10.1186/s40064-016-1780-x) contains supplementary material, which is available to authorized users.

## Background

Maize (*Zea mays* L.) is one of the most important cereals grown worldwide. Maize is an important crop in Mexico due to cultural consumption habits and economic profitability. *Fusarium verticillioides* (*Fv*) (Sacc.) Nirenb. is the most commonly reported fungal species infecting maize, causing stalk, ear and root rot (SERR) of maize, and is responsible for important economic losses worldwide (Hernández-Rodríguez et al. 2008). Maize monoculture has provoked a high incidence of the disease as well as crop losses due to *Fv*, in Mexico’s Sinaloa state (Quintero-Benítez and Apodaca-Sánchez 2008). A consortium of four different *Fusarium* species (*Fv*, *F*. *nygamai*, *F*. *thapsinum* and *F*. *andiyazi*) belonging to the *Fusarium fujikuroi* species complex (FFSC) is responsible for stalk and ear rot of maize, a current problem of maize in northern Sinaloa, Mexico (Leyva-Madrigal et al. 2015). In addition to its effects on grain yield, the infection can be detrimental to grain quality (Czembor et al. 2015).

A more complete understanding of the microbial ecology and diversity associated with the maize rhizosphere could improve plant health in field crops, reduce our dependence on chemical pesticides used in agriculture, and develop efficient biological control strategies (Filion et al. 2004). The control of pathogens by sustainable agronomic practices (such as the use of biological antagonists) has recently been adopted on a commercial scale, and a number of experimental approaches are being developed (Souza et al. 2015). Plant growth-promoting rhizobacteria (PGPR) are a heterogeneous group of bacteria that can be found in the rhizosphere, at the rhizoplane or in association with roots, and can improve the extent or quality of plant growth directly or indirectly (Ahmad et al. 2008). The following genera of bacteria have been reported as PGPR: *Agrobacterium*, *Arthrobacter*, *Azoarcus*, *Azotobacter*, *Azospirillum*, *Bacillus*, *Burkholderia*, *Caulobacter*, *Chromobacterium*, *Enterobacter*, *Erwinia*, *Flavobacterium*, *Klebsiella*, *Micrococcous*, *Rhizobium*, *Pantoea*, *Pseudomonas* and *Serratia* (Bruto et al. 2014; Ahemad and Kibret 2014) which have shown potential as biocontrol agents against different fungal pathogens (Ahemad and Kibret 2014).

Seed dressing with biocontrol agents is an appropriate method to suppress plant pathogens in the spermosphere and rhizosphere (Pereira et al. 2007). In recent years, bacterial inoculants have been used to antagonize soil-borne plant pathogens such as *Fv* and to promote plant growth. *Bacillus subtilis* and *Pseudomonas cepacia* have been used to control root rot caused by *Fv* in Argentina (Cavaglieri et al. 2005b). *Bacillus amyloliquefaciens* or *Microbacterium oleovorans* can reduce the fumonisin content in harvest grains during three evaluated seasons (Pereira et al. 2011). *Burkholderia* spp. stimulate plant growth and suppress disease caused by *Fv* in maize (Hernández-Rodríguez et al. 2008), and species like *Bacillus amyloliquefaciens* and *Enterobacter hormaechei* reduce the *Fv* infection and fumonisin accumulation in maize kernels (Pereira et al. 2010).

Biological control may result in an effective strategy for *Fv* control, but it requires the development of control microorganisms that are native to the soils where maize is grown (Etcheverry et al. 2009). The introduction of a large quantity of “exotic” microorganisms may disrupt a local ecosystem and produce ecological impacts on the rhizosphere microbiota (Jackman et al. 1992). Furthermore, microbial control agents, once released, might not only repress plant pathogens, but may also affect non-target microorganisms (Pereira et al. 2009).

This work involves the massive screening of a collection of 11,520 rhizospheric bacterial isolates obtained from both symptomatic and asymptomatic SERR plants by the use of culture media that select for specific groups previously reported as *Fv* antagonists. Based on previous knowledge on the biology of the plant-fungus and bacteria-fungus interaction and the nature of bacteria antagonistic to *Fv*, we directed the screening procedure to learn if this novel strategy would allow us to enhance our chances for finding novel native rhizospheric bacterial isolates with potential biotechnological application for the control of SERR in maize. The aims of this study were to find novel potential *Fv* biocontrol agents, and to enhance our understanding of their plant growth-promoting and antagonistic activities.

## Results

### Selection of microorganisms with an antagonist effect on *Fv*

The bacterial collection, comprising 11,520 isolates, exhibited 95 % survival efficiency 2 months after freezing and thawing, yielding a new total of 10,944 isolates. A screen of the total bacterial collection was performed using a high throughput liquid assay (PDB) method (Figueroa-López et al. 2013). We thus selected 622 isolates showing 53–99 % *Fv* growth inhibition (Additional file 1: Table S1). The main bacterial genera exhibiting an *Fv* antagonistic effect were *Bacillus* (341 isolates), *Enterobacter* (38), *Pseudomonas* (23), and *Lysinibacillus* (13), followed by members of *Acinetobacter*, *Agrobacterium*, *Anaerobranca*, *Aquaspirillum*, *Arthrobacter*, *Brevibacillus*, *Geobacillus*, *Klebsiella*, *Paenibacillus*, *Pantoea*, *Stenotrophomonas and Terribacillus* (Additional file 1: Table S1).

The selection process included a second screening test based on the same principle of a conventional dual culture in solid medium in Petri dishes, but performed in 0.2 ml 96-well plates (Figueroa-López et al. 2013). Forty-two out of the 622 selected bacterial isolates from the *Fusarium* antagonist collection displayed 45–85 % *Fv* growth inhibition in PDA (Table 1**)**. The criteria for selecting the most viable antagonists in this screening test were very stringent, only these 42 isolates that demonstrated on two independent experiments that three out of three replicates clearly showed an antagonistic effect against *Fv* were considered as viable to continue with the selection process. Most isolates belong to the genus *Bacillus* (34 isolates). This was represented by the most prominent groups or species: *B*. *cereus* sensu lato (21 isolates), *B*. *megaterium* (6 isolates) and *B*. *subtilis* group (5 isolates) (Table 1). Hemolytic tests were performed to discard isolates with possible pathogenic effect in humans, on the basis of their ability to produce hemolysins. Six isolates showed slight partial hemolysis or α-hemolysis (*B*2, *Ps*3, *B*5, *B*7, *B*12 and *B*13), and 8 isolates were non-hemolytic or γ-hemolytic (*B*4, *Pa*8, *B*9, *B*22, *B*23, *B*24, *B*25 and *B*35) (Table 1). The 28 isolates exhibiting β-hemolysis (total hemolysis) were discarded. The remaining selected isolates were used for the sterile *in planta* assay, to test for their antagonistic behavior in the presence of the host plant.

**Table 1:** Percentage of *Fv* growth inhibition in the liquid medium (PDB) and dual culture (PDA) assays, as well as hemolysis type, for the 42 isolates selected in the solid antagonistic assay yielding ≥45 % *Fv* growth inhibition

Name	Isolates	% Inhibition in PDB	% Inhibition in PDA	Hemolytic type
*B*1^a^	*Bacillus megaterium*	87	60	β
*B*2	*Bacillus megaterium*	88	66	α
*Ps*3	*Pseudomonas putida*	84	67	α
*B*4	*Bacillus flexus*	89	71	γ
*B*5	*Bacillus megaterium*	91	66	α
*B*6	*Bacillus subtilis* group	66	73	β
*B*7	*Bacillus megaterium*	74	71	α
*Pa*8	*Paenibacillus polymyxa*	62	85	γ
*B*9	*Bacillus cereus* sensu lato	62	70	γ
*B*10	*Bacillus cereus* sensu lato	83	57	β
N11	N/D	84	56	β
*B*12	*Bacillus subtilis* group	79	49	α
*B*13	*Bacillus subtilis* group	86	63	α
*B*14	*Bacillus cereus* sensu lato	81	62	β
*B*15	*Bacillus cereus* sensu lato	80	63	β
*B*16	*Bacillus subtilis* group	81	69	β
*B*17	*Bacillus subtilis* group	82	67	β
*B*18	*Bacillus cereus* sensu lato	85	82	β
*B*19	*Bacillus cereus* sensu lato	71	72	β
*B*20	*Bacillus cereus* sensu lato	85	72	β
*B*21	*Bacillus cereus* sensu lato	82	64	β
*B*22	*Bacillus megaterium*	74	68	γ
*B*23	*Bacillus megaterium*	64	63	γ
*B*24	*Bacillus cereus* sensu lato	72	73	γ
*B*25	*Bacillus cereus* sensu lato	93	52	γ
*B*26	*Bacillus cereus* sensu lato	74	58	β
N27	N/D	75	47	β
*B*28	*Bacillus cereus* sensu lato	79	52	β
*B*29	*Bacillus cereus* sensu lato	72	63	β
*B*30	*Bacillus cereus* sensu lato	83	60	β
*B*31	*Bacillus cereus* sensu lato	75	49	β
*B*32	*Bacillus cereus* sensu lato	90	45	β
*B*33	*Bacillus cereus* sensu lato	76	60	β
*B*34	*Bacillus cereus* sensu lato	86	64	β
*B*35	*Bacillus* sp.	76	73	γ
*B*36	*Bacillus cereus* sensu lato	85	70	β
N37	N/D	80	76	β
*B*38	*Bacillus cereus* sensu lato	81	69	β
N39	N/D	86	71	β
N40	N/D	95	69	β
*B*41	*Bacillus cereus* sensu lato	89	77	β
*Ps*42	*Pseudomonas fluorescens*	74	49	β

*N/D* not determined

^a^Letters preceding the isolate numbers indicate the genus of that particular isolate. *B*, N, *Ps* and *Pa* refer to *Bacillus*, not determined, *Pseudomonas* and *Paenibacillus* respectively

#### In planta assays

##### Sterile sand assay

**Fig. 1:**
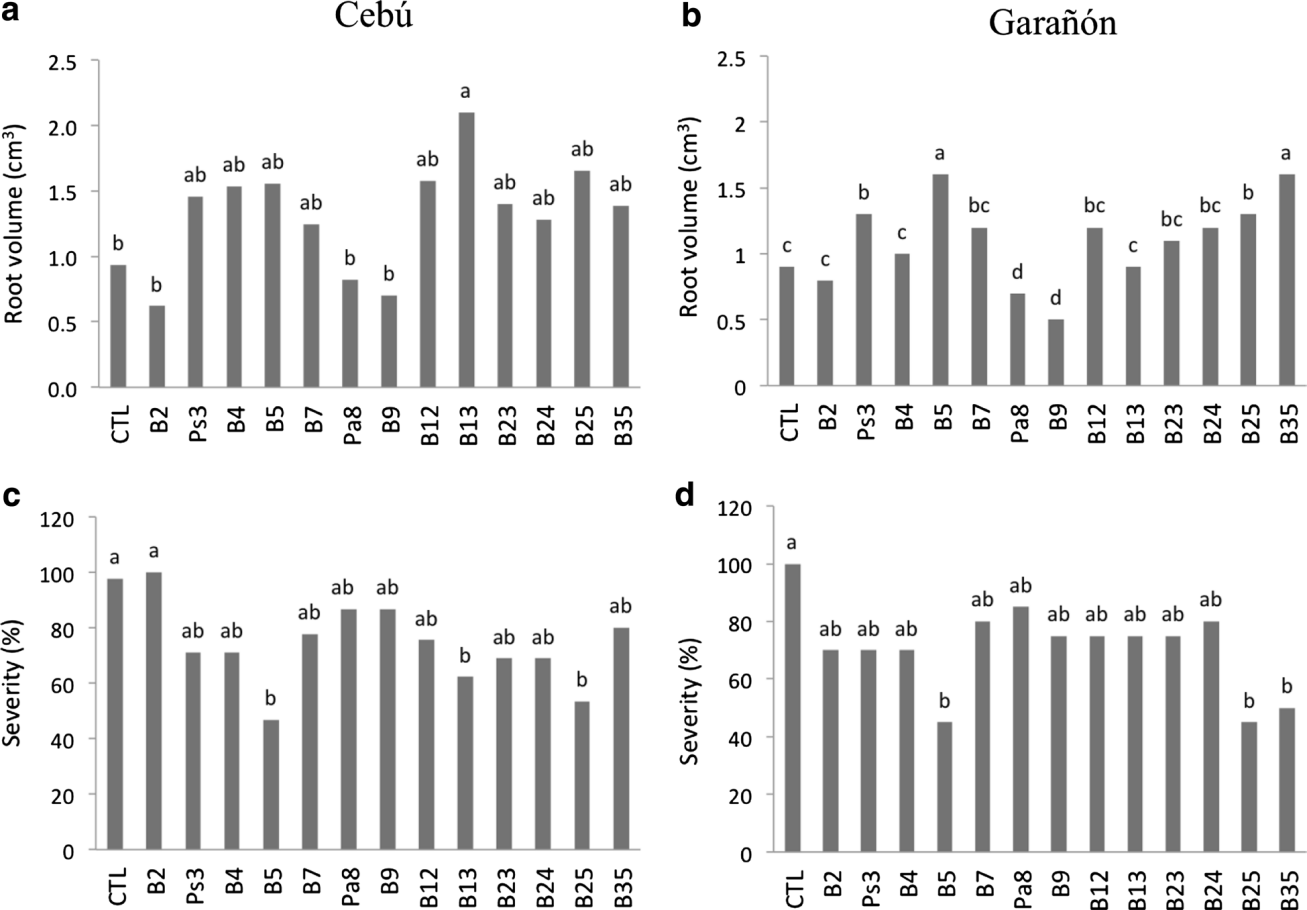
*In planta* antagonistic assays 45 days after seed emergence in two white maize hybrids inoculated with 14 partial or non-hemolytic bacterial isolates and *Fv*. **a** Root volume (Cebú hybrid), comparing a water control against the addition of the bacterial isolate. **b** Percentage of disease severity (Cebú hybrid), comparing a water control against the addition of the bacterial isolate. **c** Root volume (Garañón hybrid), comparing a water control against the addition of the bacterial isolate. **d** Percentage of disease severity (Garañón hybrid), comparing a water control against the addition of the bacterial isolate. CTL refers to the fungus control (plant plus *Fv*). Letters preceding a number indicate the genus of that particular isolate: *B* refers to *Bacillus*, *Ps* is *Pseudomonas* and *Pa* is *Paenibacillus*. Identical letters appearing above bars indicate no significant differences, while *different letters* indicate significant differences (Tukey P ≥ 0.05)

Thirteen isolates were applied to maize plants and tested as PGPRs. Isolate *B*13 showed a significant increase (60 %) in root volume in Cebu hybrid as compared to the control inoculated with *Fv* (Fig. 1a). Isolates *Ps*3, *B*5, *B*25 and *B*35 significantly increased root volume in the Garañón hybrid as compared to the control inoculated with *Fv* (Fig. 1b).

In the Cebú white maize hybrid, isolates *B*5 (47 %), *B*13 (62 %) and *B*25 (53 %) significantly reduced *Fv* disease severity compared to control plants inoculated with *Fv* (100 %) (Fig. 1c). Conversely, isolates *B*5, *B*25 and *B*35 reduced the *Fv* disease severity by 45–50 % in the Garañón hybrid, as compared to the *Fv*-treated control (Fig. 1d). An additional experiment was run in parallel to evaluate the possible effect of bacteria on root volume. This confirmed that those isolates causing a difference in the presence of *Fv* did not induce significant differences in root volume when applied alone to the seed, as compared to untreated plants (data not shown).

Although *Ps*3, *B*5, *B*13, *B*25 and *B*35 showed plant growth promoting activity on in vitro plant assays, the *B*5 and *B*25 strains decreased disease severity on both hybrids. We selected *B*25 to conduct further greenhouse tests because it was γ-hemolytic and this reduces the possibility of this strain to become pathogenic to humans.

##### Greenhouse experiments

*Fusarium* natural inoculum in soil was ~2 × 10^6^ c.f.u./g and it increased after soil inoculation in treatments with *F*. *verticillioides* P03 to ~6 × 10^7^ c.f.u./g. Incidence of *Fusarium* root and stalk rot was significantly reduced by strain *B*25 (Table 2). Stalk rot measured as percentage of severity decreased significantly when inoculated with B25 compared to both controls: untreated and *Fv* inoculated, while root rot severity decreased significantly with respect to the untreated control treatment but not to the *Fv* treated control plants (Table 2).

**Table 2:** Effect of B25 seed bacterization on *Fusarium* stalk and root rot incidence and severity on Dk2038 maize hybrid 40 days post-inoculation inoculated with *Fv* P03 in a greenhouse pot assay

Maize hybrid	Treatments	Root		Stalk	
		Incidence (%)	Severity (%)	Incidence (%)	Severity (%)
DK2038	Untreated control	100	25^a^	100	16.67^a^
	Strain B25	60	10^b^	40	6.67^b^
	Control *Fv*	100	16.66^ab^	100	16.67^a^
	Strain B25 + *Fv*	40	6.66^b^	20	3.33^b^

Different letters indicate significant differences (Duncan P < 0.05)

Non-sterile soil was used in all treatments as substrate

### Plant growth-promoting and antagonistic traits of bacterial isolates tested *in planta*

To explore which mechanisms of growth promotion could be involved in *Fv* control, we examined different antagonistic traits in the isolates evaluated in the maize antagonistic assays (Table 3). Phosphate solubilization was detected in isolates *B*4, *B*5, *Pa*8, *B*12, *B*13 and *B*23. IAA production was shown only for *Pa*8, which produced 40 µmol/l of auxin-like compounds. Siderophores were produced by isolates *Ps*3, *B*4, *B*5, *B*7, *B*12, *B*13, *B*22, *B*24 and *B*25. Protease activity was present in *B*4, *B*5, *B*7, *B*12, *B*22, *B*24 and *B*25. Chitinase activity was displayed by isolates *B*13, *B*23, *B*24 and *B*25. All isolates exhibited glucanase activity except for *B*12 and *B*23 (Table 3).

**Table 3:** Plant growth promotion and antagonistic traits of the 14 isolates selected as partial- or non-hemolytic

Name^a^	Isolates	Phosphate	Auxin	Siderophore	Chitinase	Glucanase	Protease
*B*2	*Bacillus megaterium*	−	−	−	−	+	−
*Ps*3	*Pseudomonas putida*	−	−	+	−	+	−
*B*4	*Bacillus flexus*	+	−	+	−	+	+
*B*5	*Bacillus megaterium*	+	−	+	−	+	+
*B*7	*Bacillus megaterium*	−	−	+	−	+	+
*Pa*8	*Paenibacillus polymyxa*	+	+	−	−	+	−
*B*9	*Bacillus cereus* sensu lato	−	−	−	−	+	−
*B*12	*Bacillus subtilis group*	+	−	+	−	−	+
*B*13	*Bacillus subtilis group*	+	−	+	+	+	−
*B*22	*Bacillus megaterium*	−	−	+	−	+	+
*B*23	*Bacillus megaterium*	+	−	−	+	−	−
*B*24	*Bacillus cereus* sensu lato	−	−	+	+	+	+
*B*25	*Bacillus cereus* sensu lato	−	−	+	+	+	+
*B*35	*Bacillus* sp.	−	−	−	−	+	−

^a^Letters preceding the numbers of the isolate indicate the genus of that particular isolate. *B* refers to *Bacillus*, *Ps* to *Pseudomonas* and *Pa* to *Paenibacillus*. (+) Indicates a positive result, (−) indicates a negative result for each specific assay

## Discussion

The effect of a pathogen on native microbial communities has previously been studied in diseases affecting crops besides maize, such as citrus (Trivedi et al. 2010; Araujo et al. 2002), conifers (Filion et al. 2004), wheat (McSpadden Gardener and Weller 2001), potato (Reiter et al. 2002), and avocado (Yang et al. 2001). These studies demonstrate that phytopathogens affect both endophytic and rhizospheric bacteria populations, and that some bacterial populations may assist the plant in preventing disease symptoms, by inhibiting phytopathogen growth.

The aim of our work was to find bacterial isolates that control *Fv* growth. Our approach, a fast high-throughput screening liquid antagonistic assay coupled to a conventional dual-culture antagonism assay, allowed identifying such isolates that could be potential *Fv* antagonists. The rationale for using both screening methods was that the first screening could select not only for diffusible substances produced by the bacterial isolates affecting *Fv* growth but also for organisms that could affect fungal growth only when they enter in contact with the fungus, the dual-culture antagonism assay should then confirm which of those selected isolates have the best antagonistic effect against *Fv* before entering in contact with the fungus. The main reason for executing the screening in this order was that the liquid screening is less time-consuming than the dual-culture antagonism assay. It is possible that by performing the screening in this order we had missed a few isolates that are effective controlling *Fv* growth by entering in contact with the fungus. Using different types of antagonism assays during a large screening like the one performed in this work should allow selecting for isolates that employ different biocontrol strategies.

Massive screening assays designed to find potential antagonistic bacteria against fungal phytopathogens have limitations proper of the specific test. The initial screening step performed on PDB was biased to favor *Fv* growth. Although preliminary tests (Figueroa-López et al. 2013) allowed us to learn that most bacterial isolates would be able to grow well in PDB, we found bacteria that either did not show growth at all (>2.5 %), or did exhibit visually poor growth in PDB (>1 %) (data not shown). These bacteria probably will not be able to produce enough of the substances that may inhibit *Fv* growth and will not reach a threshold that will allow them to exhibit an antagonistic response. Thus, it is possible that our initial screening assay may have left aside a few potential isolates that were unable to perform well under these assay conditions.

Different species of *Bacillus* represented more than half of the identified *Fv* antagonistic isolates (Additional file 1: Table S1), as well as the most prominent bacterial populations in this study. Different species belonging to all other genera that showed fungal inhibition in liquid assay (Additional file 1: Table S1) including *Acinetobacter* (Magnin-Robert et al. 2007), *Arthrobacter* (Cavaglieri et al. 2005a), *Enterobacter* (van Dijk and Nelson 1997; Gopalakrishnan et al. 2011), *Klebsiella* (Lynch 1990), *Lysinibacillus* (Trivedi et al. 2011), *Paenibacillus* (Nielsen and Sorensen 1997), *Pantoea* (Babalola 2010) and *Pseudomonas* (Gorlach-Lira and Stefaniak 2009) are reported to possess antagonistic traits against diverse pathogens.

*Pseudomonas putida* and *P*. *fluorescens* inhibited *Fv* growth in the solid medium assay (Table 1). Different *Pseudomonas* spp. produce a broad array of lytic enzymes, antibiotics, cyanide, siderophores and antifungal compounds (Nagarajkumar et al. 2004; Weller et al. 2007; Gorlach-Lira and Stefaniak 2009) which can all inhibit pathogens including *Fv*.

The *Bacillus* genus is able to produce many secondary metabolites with antifungal effects on diverse plant pathogens (Raaijmakers and Mazzola 2012). Cavaglieri et al. (2005a) demonstrated the antagonistic effect of ten *Bacillus* isolates (including *B*. *cereus*) against *Fv* with growth inhibition percentages ranging from 28 to 78 %; by comparison, our study revealed percentages between 45 and 85 % in solid medium (Table 1). *Bacillus* species use diverse mechanisms that may inhibit this fungal pathogen (Ongena and Jacques 2008) including nutrient competition (Kamilova et al. 2005), production of antifungal lipopeptides (Nihorimbere et al. 2012), or production of lytic enzymes such as chitinases that can degrade the fungal cell wall as a means to avoid fungal hyphal extension (Kishore et al. 2005). The stringent conditions used in the dual-plate assay reduced significantly the amount of isolates from 622 to only 42. Nevertheless, we cannot discard the possibility that by doing this we had missed some isolates that could be effective in the next selection steps. Time and space constraints for performing *in planta* assays, either in growth chambers or greenhouses, constitute important factors for selecting fewer isolates to work with in plant species such as maize.

*In vitro* testing of *B*. *subtilis* in maize roots and kernels has previously been reported to inhibit both *Fv* growth and the production of fumonisin B1 (Cavaglieri et al. 2004). Nevertheless, results obtained in vitro are not necessarily reproducible when the host plant is included in its tripartite interaction with the fungus and the bacterium. This is probably due to the plant exudates, which could affect the fungus and/or bacteria in different ways (Fan et al. 2012). Furthermore, different results may be obtained when the potential bacterial antagonist is placed under field conditions and other biotic or abiotic factors are considered (Egamberdiyeva 2007). On the other hand, satisfactory results using endophytic *B*. *subtilis* in maize causes a reduction in mycotoxin production and a decrease in *Fv* colonization (Bacon et al. 2001). *Bacillus cereus* increased grain yield by 43.8 % in maize (Tilak and Reddy 2006).

Application of bacteria to seeds (bacterization) has been widely used for the biological control of soil-borne plant pathogens that affect many host plants (Cavaglieri et al. 2005a). In this work, we used plant-based experiments to evaluate 13 native isolates of the maize rhizosphere that are capable of inhibiting *Fv* growth in vitro. Our results suggest a beneficial response in growth promotion (measured as root volume), as well as on *Fv* disease severity between some isolates and the white maize hybrids tested (Fig. 1). *Bacillus megaterium* (*B*5) and *B*. *cereus* sensu lato (*B*25) reduced *Fv* disease severity in both white maize hybrids tested. *Bacillus megaterium* has been reported to act as a biocontrol agent of *Fusarium* crown and root rot of tomato (Omar et al. 2006).

We selected B25 to conduct greenhouse pot experiments based on its lack of hemolytic activity and ability to decrease *Fv* disease severity in both white maize hybrids tested (Garañón and Cebú) under sterile in vitro conditions. Many human pathogenic bacteria produce soluble proteins that can lyse erythrocytes (Herlax and Bakas 2002), the *B*25 strain was γ-hemolytic (no-hemolysis) suggesting that it is not harmful to human health. *B*25 was assayed under non-sterile conditions and a third white maize hybrid (DK2038 from Dekalb) commonly employed in northern Sinaloa, Mexico.

*B*25 reduced the disease incidence and in most cases disease severity confirming the in vitro results and suggesting a good capability to establish well in the rhizosphere and compete against other rhizospheric microorganisms present in the soil. This effect could be due to the different plant growth promotion activities that *B*25 possesses (Table 3).

Another focus of our study was plant growth-promoting or antagonistic traits used by isolates to inhibit *Fv* growth and decrease disease severity in maize plants. We investigated these traits by performing several tests and found that isolates use different mechanisms (Table 3). Phosphate solubilizing bacteria, including *Bacillus* species (Kumar et al. 2012), can play an important role in plant nutrition, by increasing phosphorus uptake in plants (Rodríguez et al. 2007). The siderophores produced by PGPRs are capable of inhibiting root pathogens by creating limiting iron conditions in the rhizosphere. The *Bacillus* isolates *B*5, *B*13 and *B*25 reported in this work are siderophore producers and potential biocontrol agents (Table 3), which are able to reduce the disease severity in maize plants (Table 2; Fig. 1). One *Bacillus subtilis* strain has been reported to produce bacilibactin and itoic acid, as well as siderophores, and is able to reduce *Fusarium* wilt incidence in pepper (Yu et al. 2011). On the other hand, isolates *B*5 and *B*25 were observed to exhibit protease activity. These antifungal proteins are responsible for inhibition against diverse pathogens including *Fv* in this work, and other fungi including *F*. *oxysporum*, *F*. *solani*, *P*. *ultimum* and *Rhizoctonia solani* (Chang et al. 2008; Gao et al. 2008). In this study, isolates of *B*. *cereus* sensu lato (*B*24 and *B*25), *B*. *subtilis* group (*B*13) and *Bacillus megaterium* (*B*23) showed chitinase activity, suggesting that it could be a potential trait involved in *Fv* growth control for these isolates (Bressan and Fontes-Figueiredo 2010). Production of chitinases by *Bacillus* was demonstrated to cause a reduction in *F*. *graminearum* infection in wheat (Shali et al. 2010). Chitin and glucan are the main structural components of the fungal cell wall (Yang et al. 2004), and most isolates (including *B*5, *B*13, *B*25 and *B*35) produced glucanases. The observed chitinase and glucanase activities suggest that they may be implicated in the inhibition and reduction of disease severity by all isolates producing these enzymes.

Some bacteria may share similar repertoires of hydrolytic enzymes or antagonistic traits when tested in vitro, but the results may differ when the other participants in this interaction (i.e. the plant and the fungus) intervene. This is suggested by the observation that the pairs *B*4 and *B*5 or *B*24 and *B*25 share similar activities, despite the fact that no *Fv* control was exerted by *B*4 or *B*24. Although the underlying reasons are currently not understood, our research group is currently conducting work in parallel to elucidate the mechanisms used by these bacteria to exert biocontrol *in planta*.

Special consideration was given to the discovery of native bacterial isolates in this work, since such biocontrol agents could be well suited to edaphic and climatological conditions from a specific region. Additionally, co-existence for many years with the natural soil microbiota should provide native microorganisms with competitive advantages compared to exotic species. The research presented here was aimed at identifying native biocontrol agents and bacteria capable of exerting biocontrol against *Fv*, as well as providing valuable information on the agriculturally natural conditions of these bacterial populations in maize plants. Only cultivable microorganisms can be able to develop bio-fertilizers against crops diseases (Jha et al. 2010) and the native microorganism are promising alternative. Our findings with *Fv* antagonistic assays in vitro and *in planta* have been recently confirmed in field trials (Lizárraga-Sánchez et al. 2015) and the efficacy of the isolates have been proved in order to exploit them as potential biocontrol agents suitable for widespread use in the large extensions of maize sown in Sinaloa, Mexico.

## Conclusions

In this study we analyzed a large number of microorganisms using an easy and time-saving in vitro screening method that allowed us to find a potential biocontrol agent in a short amount of time. We suggest for future studies concerning the selection of bacterial antagonists against fungal plant pathogens that before designing a protocol for massive screening assays, decisions should be taken based on: (1) the best available knowledge on the biology of the specific plant-fungal interaction both in vitro and under field conditions; (2) the possible nature and source of the isolates; (3) the best isolation and assay culture media; (4) the relative abundance of the potential antagonists in the rhizosphere microbiota; and (5) as many factors as possible to plan the screening protocol. Screening of a collection of 11,520 rhizospheric bacteria allowed identifying three *Bacillus* isolates (*B*5, *B*25, *B*35) as potential antagonists to inhibit maize rots caused by *Fv*. These strains produce lytic enzymes such as glucanases, proteases or chitinases, as well as siderophores and auxins and suggests these as possible control mechanisms against *Fv*. *Bacillus cereus* sensu lato *B*25 was selected to conduct greenhouse pot assays that showed a reduction of *Fv* disease severity and incidence on plants confirming the potential of this isolate to control *Fv* in maize. *B*25 strain was effective in controlling *Fv* possibly due to the several PGPR traits that the bacterium possesses. *B*25 is currently being studied to develop a novel biological product based on spore production (Martínez-Álvarez et al. 2016) to reduce *Fusarium* stalk, ear and root rots in maize fields.

## Methods

### Sample collection

A total of fifty maize rhizosphere samples were collected from five locations in Sinaloa state (Mexico): (1) Serrano; (2) Alhuey; (3) 18 de Diciembre; (4) Casa Blanca; and (5) La Trinidad. These fields showed symptoms of plant damage by the fungus *Fv*. Plants were sampled in five paired groups, each pair consisting of one symptomatic plant and one asymptomatic plant grown side by side. The five samples from each condition and location were homogenized together and stored at room temperature. 3–4 kg of bulk soil were removed from the stem base of each plant. Each of the five sampling points differed by planting day and by maize hybrids. Microbiological analyses were conducted to confirm SERR symptomatology, and *Fv* was isolated from SERR symptomatic plants in selective media.

### Sample processing

Soil particles adhering to the roots (rhizospheric soil) were collected and microorganisms were then isolated by serial dilutions. Four different culture media were prepared in 100 mm-diameter Petri dishes to enrich for specific taxonomic groups: Luria Bertani (LB) medium was used to enrich for *Bacillus* isolates (Cavaglieri et al. 2005a); Actinomycetes Isolation Agar (AIA) was used for Actinomycetes isolates (Bressan and Fontes-Figueiredo 2007); King B Agar (KBA) was used for *Pseudomonas* (Cavaglieri et al. 2004); and Man, Rogosa and Sharpe (MRS) medium was used for lactic acid bacteria (De Man et al. 1960). Colonies were collected from LB, KBA and MRS media after 24 h growth and from AIA medium after 48–72 h at 25 °C. A bulk soil sub-sample (500 g) was used for nutrient and physicochemical soil analyses.

### Microorganism collection and viability test

To generate the maize rhizospheric culturable bacterial collection, 288 isolates were “picked” and arranged in three 96-well plates from each specific culture medium. This was performed for each composite sample with the five symptomatic-rhizosperic samples, as well as the five asymptomatic rhizosperic samples per each maize field. This yielded 1152 isolates from each of the ten composite rhizospheric sample points; the complete collection therefore contained 11,520 isolates. Isolates were cryopreserved in triplicate at −70 °C, using LB containing 15 % glycerol (v/v) according to Pasarell and McGinnis (1992). Frozen stocks were made and grown at 25 °C in rotary shaker at 200 rev/min in 2 ml 96-well plates containing 1.5 ml liquid medium, for either 24 h (LB, KBA and MRS media) or 72 h (AIA medium). The isolate was considered non-viable if no visible growth was observed after thawing. Plates containing bacterial pellets from viable isolates were stored at −70 °C until processing for DNA extraction.

### Molecular identification of bacteria using 16S rDNA

Bacterial DNA was extracted with the DNeasy^®^ Blood & Tissue Kit (Qiagen; CA, USA). The primers F2C (5′-AGAGTTTGATCATGGCTC-3′) and C (5′-ACGGGCGGTGTGTAC-3′) (Shi et al. 1997) were used to amplify 16S rDNA. PCR reactions were carried out in 96-well plates. The 25 μl PCR mixture contained 10 ng of DNA template, 1X reaction buffer, 10 pmol of each primer, 10 µmol/l of each deoxynucleoside triphosphate (dNTP), and 1 U of Taq DNA polymerase (Invitrogen; Carlsbad, CA, USA). The PCR conditions included an initial denaturation step at 95 °C (4 min); 32 cycles of denaturation at 95 °C (1 min) followed by annealing at 60 °C (5 min) and extension at 72 °C (1.5 min); and a final step at 72 °C (5 min). The PCR was performed using a MyCycler thermal cycler (BioRad; CA, USA). Products were visualized by 1 % agarose (w/v) gel electrophoresis in 0.5 X Tris–acetate-EDTA (TAE) buffer and stained with ethidium bromide. PCR products were purified with a QIAquick PCR Purification kit (Qiagen; CA, USA) and quantitated using a Nanodrop 2000 UV–Vis spectrophotometer (Thermo Scientific). The U1 primer (5′-CCAGCAGCCGCGGTAATACG-3′) (Lu et al. 2000) internal to the F2C/C amplified PCR product was used for sequencing with an ABI 3730 XL automated sequencer at the National Laboratory of Genomics (LANGEBIO; Irapuato, Mexico). Isolates were identified by sequence comparison against the Ribosomal Database Project (RDP) and GenBank databases using the BLASTN search algorithm (http://blast.ncbi.nlm.nih.gov). The sequences were then compared on the basis of identity percentage, E-value and Match score, using the default parameters from the RDP seq match tool.

### Screening of *Fv* antagonists

The antagonism selection assay was performed to analyze all viable bacterial specimens from the collection, according to (Figueroa-López et al. 2013). Selection was performed in two steps. First, a liquid antagonism assay using potato dextrose broth (PDB) was performed. Briefly, this consisted of growing the isolate and the fungus together in 2 ml 96-well plates and quantifying the fungal biomass by staining the chitin residues of the fungal cell wall with the wheat germ agglutinin lectin coupled to a fluorophore (WGA Alexa Fluor 488 conjugate), which was then measured using a multimodal fluorescence detector (Beckman, DTX800). The selection criterion was arbitrarily set at >50 % fungal growth inhibition. The bacterial isolates derived from this screen were assayed in a second selection step using regular dual antagonism assays with 0.2 ml of potato dextrose agar (PDA) medium in 96 well plates to confirm the effect observed in the first assay. In this second assay, the selection criterion was set for isolates to display ≥45 % fungal growth inhibition in all three replicates.

### Blood hemolytic assay

Hemolysis tests were performed in order to discard isolates that could be pathogenic to humans. Bacterial isolates were grown in 15 ml tubes containing 5 ml of LB at 25 °C for 24 h in a rotary shaker at 250 rev/min. One ml of bacterial culture was taken and transferred to a 1.5 ml tube, centrifuged twice at 16,800*g* for 5 min, and the resulting supernatant was transferred to a new tube. 5 mm-diameter wells were made in blood agar plates with a cork borer, and 50 µl of supernatant were aliquoted into the wells. The plates were then stored at 37 °C for 24 h. Complete β-hemolysis was observed as a clear zone around the well in the blood agar medium, indicating complete breakage of erythrocytes. Conversely, partial α-hemolysis was observed as a dark-green coloration around the well, indicating the partial damage of erythrocytes. Bacteria with γ-hemolysis do not exhibit any alteration of color or opacity in the medium, indicating an absence of hemolysis (Forbes et al. 2002).

### *In planta* antagonistic assays

#### Sterile sand assay

Two types of white maize hybrid seeds, Cebú and Garañón (Asgrow), were used for the *in planta* antagonism assay. Seeds were surface-sterilized prior to bioassays by placing them in 0.75 % sodium hypochlorite (w/v) at 52 °C for 20 min, followed by three copious washes with sterile distilled water for 5 min each (Daniels 1983). This methodology yielded 98–100 % seed germination with 1–3 % seed contamination. For this reason, seeds were pre-germinated on Komada’s *Fusarium*-selective medium (Komada 1975), and seeds with no symptoms of fungal growth (i.e. *Fv*-free) were selected, whereas those presenting contamination were eliminated. Bacterial isolates were grown in 15 ml tubes containing 5 ml of LB medium at 25 °C for 24 h at 250 rev/min, and an optical density (OD) of 1.0 at 595 nm was used to calculate the colony-forming units (c.f.u./ml) after plating. Maize seeds were soaked in bacterial suspensions containing 1.5 × 10^8^ c.f.u./ml for 20 min. Three seeds were planted per sterile polypropylene container (similar to a Magenta box) containing 200 g of wet sterile sand. Bacterial-treated seeds were transferred to sand inoculated with *Fv* isolate P03 2 days before sowing, and control untreated seeds were placed in sterile sand containing *Fv* (which was added at a concentration of 1 × 10^5^ conidia/g of sand). Nine plants per control or treatment were evaluated in three experimental sets containing three seeds each. The experiment was evaluated 45 days after seed emergence. Root volume was measured according to Burdett (1979) and disease severity was evaluated as described in Cumagun et al. (2009). An additional experiment was performed as a control to confirm that bacteria did not cause any detrimental effects to root volume; this experiment used a set of bacterial-treated seeds compared to an untreated seed control (receiving water) grown in sterile sand under the same conditions as listed above (data not shown).

#### Greenhouse assay

*Fv* isolate P03 was reactivated on PDA plates by incubation at 25 °C for 14 days. Three mycelial plugs (7 mm diameter) were transferred to sterile plastic bags containing 100 g of sterile cracked maize, hydrated with 40 ml of sterile distilled water and incubated at 25 °C for 14 days (Leyva-Madrigal et al. 2015). Sixty-five grams of the maize/fungus mix was then added to 1 kg styrofoam pots containing a mixture of non-sterile vermiculite:non-sterile soil (1:1 v/v). Fungal inoculum concentration (c.f.u./g) was estimated using the “massive stamping drop plate” method (Corral-Lugo et al. 2012) in Nash–Snyder agar plates. To inoculate plants with bacteria, root systems of five-days-germinated plantlets (hybrid Dk2038 from Dekalb^®^) were submerged for 10 min in a bacterial suspension (5 × 10^8^ c.f.u/ml) of B25, blot dried and then planted in a pot. One plant was placed per pot (one replicate). A completely randomized design with 5 replicates per treatment was used. The following treatments were included: untreated control (untreated maize seeds); strain B25 (maize seeds treated with *B*. *cereus* B25 without *Fv* P03); control *Fv* (untreated maize seeds containing *Fv* P03); strain B25 + *Fv* (maize seeds treated with *B*. *cereus* B25 and *Fv* P03).

Pots were kept in greenhouse conditions at 28 ± 2 °C with a natural photoperiod for 40 days, where they were watered two times a week with distilled water. Disease incidence was evaluated by visual assessment and reported as positive if the presence of any signs of disease was observed. Visual disease symptoms on roots were assessed according to the severity scale reported by Soonthornpoct et al. (2000). Data were converted to a disease index score using the formula reported by Asran and Buchenauer (2003).

### Characterization of functional plant growth-promoting and/or antagonistic traits of bacterial isolates

Plate screening assays were used to investigate plant growth-promoting and antagonistic traits. Auxin production was evaluated using Salkowsky’s reagent (Loper and Schroth 1986). Briefly, single colonies were grown in LB broth for 24 h and the supernatants were treated with Salkowsky’s reagent, according to Bric et al. (1991). After several minutes, IAA production was identified by a color change in the supernatant. Isolates were streaked and screened for phosphate-solubilizing ability on Pikosvkaya’s agar (Pikosvkaya 1948). After incubation for 1 week at 25 °C, the presence of a clear zone around the bacterial colony was considered positive for phosphate solubilizing. The chitinase assay was performed on colloidal chitin agar medium according to (Shanmugaiah et al. 2008), and chitinase activity was identified by the formation of a clear zone around the bacterial cells after 5 days of growth. *In vitro* β-1, 4-endoglucanase activity was assayed using carboxy-methyl cellulose (CMC; Cat 419273, Sigma Chemicals Company; St. Louis, MO, USA) as the substrate. Single colonies were grown in LB broth for 48 h at 30 °C. Two hundred µl of cell-free supernatant was placed in 5 mm-diameter wells (previously made using a cork borer) in 1 % CMC agar plates, and incubated for 24 h at 30 °C. The formation of a clear zone around a well, resulting from β-1, 4-endoglucanase activity, was revealed by adding 5 ml of Congo red 1 % w/v for 15 min. Subsequently, the Congo red dye was removed and 5 ml NaCl 2 mol/l was added for 15 min to eliminate the excess dye, and to visualize the formation of clear zones (Teather and Wood 1982). Siderophore production was determined after 1 week of incubation in chrome azurol S (CAS) agar. The CAS blue solution for this assay was prepared according to Schwyn and Neilands (1987). Pure isolates were pricked onto CAS agar plates using sterile toothpicks and incubated at 25 °C for 2 weeks in the dark, and the assay was performed in triplicate. Colonies with yellow/orange zones were considered to be siderophore-producing strains. Protease activity was tested in skimmed milk agar (SMA) with commercially available non-fat milk, according to Jones et al. (2007). The strains were streaked onto SMA, and plates were incubated for 24 h at 30 °C. Protease activity was identified by the formation of a clear zone around the bacterial colonies. All assays were performed in triplicate for each bacterial isolate tested.

### Statistical analyses

A completely randomized experimental design was used for plant experiments. The obtained severity scale values were evaluated by a normality test, using the Shapiro Will test and a Bartlett’s test to confirm variance homogeneity. Data were parametric, and severity scale data were subjected to statistical analysis of variance (ANOVA) to detect differences between treatments. All percentage values were previously converted to arcsine [√(x %/100) + 0.5] for data normalization and to proceed with the analysis of variance (Dughetti and García 2004). Mean comparisons were made using Tukey’s and Duncan’s tests; all statistical tests were conducted at a probability level of P ≤ 0.05. All analyses were performed using the Statistical Analysis System 9.0 software (SAS Institute; Cary, NC).

## Additional file

10.1186/s40064-016-1780-x Isolates showing >50 % *Fv* growth inhibition obtained from the large-scale liquid antagonism assay, and the corresponding name of the 42 isolates (Name column) selected for their antagonistic activity in solid medium (see Table 1).

## Abbreviations

*Fv*: *Fusarium verticillioides*
SERR: stalk, ear and root rot
LB: Luria Bertani
