# Supplementary material for: Rhizospheric bacteria of maize with potential for biocontrol of Fusarium verticillioides
Source: Springerplus. 2016 Mar 15;5:330. doi: 10.1186/s40064-016-1780-x (PMC4792820; doi:10.1186/s40064-016-1780-x)
Supplement: Supplementary file 1 — 10.1186/s40064-016-1780-x Isolates showing >50 % Fv growth inhibition obtained from the large-scale liquid antagonism assay, and the corresponding name of the 42 isolates (Name column) selected for their antagonistic activity in solid medium (see Table 1). [file 40064_2016_1780_MOESM1_ESM.docx]

**Table S1**

| **List No.** | **GenBank Accesion** | **Putative species** | **Inhibition (%)** | **Name** |
| --- | --- | --- | --- | --- |
| 1 | ^3^N/A | *Bacillus megaterium* | 87 | *^1^B*1 |
| 2 |  | ^2^N/D | 67 |  |
| 3 | N/A | *Bacillus megaterium* | 88 | *B*2 |
| 4 | JQ830008 | *Bacillus megaterium* | 95 |  |
| 5 | JQ830014 | *Bacillus bataviensis* | 75 |  |
| 6 | JQ830019 | *Bacillus megaterium* | 77 |  |
| 7 | JQ830029 | *Pseudomonas putida* | 93 |  |
| 8 | JQ830047 | *Enterobacter cloacae* | 70 |  |
| 9 | JQ830055 | *Enterobacter cloacae* | 73 |  |
| 10 | JQ830057 | *Enterobacter cloacae* | 72 |  |
| 11 | JQ829806 | *Bacillus megaterium* | 91 |  |
| 12 |  | N/D | 63 |  |
| 13 | JQ829835 | *Bacillus subtilis* group | 65 |  |
| 14 | JQ829857 | *Bacillus megaterium* | 76 |  |
| 15 | N/A | *Bacillus cereus sensu lato* | 86 |  |
| 16 | N/A | *Bacillus megaterium* | 67 |  |
| 17 | N/A | *Bacillus megaterium* | 86 |  |
| 18 | JQ829897 | *Bacillus flexus* | 91 |  |
| 19 | JQ829904 | *Bacillus subtilis* group | 72 |  |
| 20 | JQ829909 | *Pseudomonas putida* | 84 | *Ps*3 |
| 21 | JQ829910 | *Bacillus subtilis* group | 90 |  |
| 22 | JQ829915 | *Bacillus flexus* | 77 |  |
| 23 | JQ829937 | *Paenibacillus lautus* | 83 |  |
| 24 | JQ829951 | *Bacillus megaterium* | 78 |  |
| 25 | JQ829965 | *Bacillus flexus* | 76 |  |
| 26 | JQ830750 | *Bacillus subtilis* group | 87 |  |
| 27 | JQ830820 | *Bacillus megaterium* | 87 |  |
| 28 | JQ830822 | *Bacillus flexus* | 89 | *B*4 |
| 29 | JQ830824 | *Bacillus flexus* | 68 |  |
| 30 | JQ830832 | *Bacillus megaterium* | 91 | *B*5 |
| 31 | JQ830833 | *Bacillus subtilis* group | 84 |  |
| 32 | JQ830840 | *Bacillus megaterium* | 72 |  |
| 33 | JQ830841 | *Bacillus megaterium* | 79 |  |
| 34 | JQ830856 | *Bacillus subtilis* group | 69 |  |
| 35 | JQ830865 | *Enterobacter cloacae* | 76 |  |
| 36 | JQ830887 | *Bacillus megaterium* | 68 |  |
| 37 | JQ830889 | *Bacillus megaterium* | 68 |  |
| 38 | JQ830897 | *Bacillus megaterium* | 66 | *B*6 |
| 39 | JQ830907 | *Bacillus niacini* | 79 |  |
| 40 | JQ830909 | *Bacillus cereus sensu lato* | 79 |  |
| 41 | JQ830911 | *Bacillus megaterium* | 75 |  |
| 42 | JQ830916 | *Bacillus megaterium* | 71 |  |
| 43 | JQ830925 | *Bacillus megaterium* | 85 |  |
| 44 | JQ830928 | *Bacillus flexus* | 81 |  |
| 45 | JQ830931 | *Bacillus cereus sensu lato* | 71 |  |
| 46 | JQ830944 | *Bacillus subtilis* group | 87 |  |
| 47 | JQ830955 | *Bacillus cereus sensu lato* | 88 |  |
| 48 | JQ830963 | *Bacillus cereus sensu lato* | 69 |  |
| 49 | JQ830964 | *Bacillus megaterium* | 69 |  |
| 50 | JQ830967 | *Bacillus cereus sensu lato* | 88 |  |
| 51 | JQ830974 | *Bacillus flexus* | 85 |  |
| 52 | JQ831553 | *Bacillus cereus sensu lato* | 72 |  |
| 53 | JQ831562 | *Bacillus endophyticus* | 66 |  |
| 54 | JQ831570 | *Bacillus megaterium* | 72 |  |
| 55 | JQ831576 | *Bacillus cereus sensu lato* | 79 |  |
| 56 | JQ831587 | *Bacillus cereus sensu lato* | 72 |  |
| 57 | JQ831600 | *Bacillus subtilis* group | 73 |  |
| 58 | JQ831601 | *Bacillus megaterium* | 72 |  |
| 59 | JQ831612 | *Bacillus megaterium* | 75 |  |
| 60 | JQ831623 | *Bacillus subtilis* group | 74 |  |
| 61 | JQ831635 | *Bacillus cereus sensu lato* | 79 |  |
| 62 | JQ831638 | *Bacillus subtilis* group | 70 |  |
| 63 | JQ831642 | *Bacillus subtilis* group | 93 |  |
| 64 | JQ831736 | *Bacillus subtilis* group | 71 |  |
| 65 | JQ831744 | *Enterobacter hormaechei* | 72 |  |
| 66 | JQ831769 | *Bacillus megaterium* | 81 |  |
| 67 | JQ831775 | *Enterobacter sp.* | 77 |  |
| 68 | JQ831778 | *Bacillus megaterium* | 74 | *B*7 |
| 69 | JQ831799 | *Bacillus megaterium* | 86 |  |
| 70 | JQ832197 | *Bacillus flexus* | 92 |  |
| 71 |  | N/D | 82 |  |
| 72 | JQ832235 | *Bacillus megaterium* | 82 |  |
| 73 | N/A | *Bacillus cereus sensu lato* | 72 |  |
| 74 | N/A | *Bacillus subtilis* group | 75 |  |
| 75 | JQ832273 | *Bacillus axarquiensis* | 72 |  |
| 76 | N/A | Sequence not found | 75 |  |
| 77 | JQ832289 | *Bacillus oceanisediminis* | 71 |  |
| 78 | JQ832292 | *Bacillus flexus* | 92 |  |
| 79 | JQ832294 | *Bacillus megaterium* | 83 |  |
| 80 | JQ832300 | *Bacillus endophyticus* | 77 |  |
| 81 | JQ832303 | *Bacillus megaterium* | 72 |  |
| 82 |  | N/D | 71 |  |
| 83 | JQ832318 | *Bacillus megaterium* | 73 |  |
| 84 | JQ832359 | *Bacillus megaterium* | 85 |  |
| 85 | JQ832391 | *Bacillus flexus* | 61 |  |
| 86 | JQ832407 | *Bacillus megaterium* | 63 |  |
| 87 | N/A | *Paenibacillus polymyxa* | 62 | *Pa*8 |
| 88 | JQ832940 | *Bacillus subtilis* group | 55 |  |
| 89 | JQ832950 | *Bacillus endophyticus* | 62 |  |
| 90 | JQ832951 | *Bacillus firmus* | 53 |  |
| 91 | JQ832967 | *Bacillus megaterium* | 65 |  |
| 92 | N/A | *Bacillus cereus sensu lato* | 62 | *B*9 |
| 93 | JQ832994 | *Bacillus flexus* | 90 |  |
| 94 | JQ833012 | *Bacillus subtilis* group | 92 |  |
| 95 | JQ833042 | *Acinetobacter lwoffii* | 92 |  |
| 96 | JQ833045 | *Bacillus megaterium* | 90 |  |
| 97 | JQ833055 | *Bacillus flexus* | 79 |  |
| 98 | JQ833056 | *Klebsiella pneumoniae* | 82 |  |
| 99 | JQ833058 | *Bacillus flexus* | 83 |  |
| 100 | JQ833076 | *Bacillus subtilis* group | 73 |  |
| 101 |  | N/D | 85 |  |
| 102 | JQ833115 | *Bacillus megaterium* | 85 |  |
| 103 | JQ833378 | *Bacillus subtilis* group | 81 |  |
| 104 | JQ833407 | *Bacillus subtilis* group | 73 |  |
| 105 | JQ833429 | *Pseudomonas lini* | 90 |  |
| 106 | JQ833437 | *Pseudomonas corrugata* | 71 |  |
| 107 | JQ833466 | *Pseudomonas lini* | 90 |  |
| 108 | JQ833494 | *Pseudomonas lini* | 88 |  |
| 109 | JQ833497 | *Pseudomonas corrugata* | 82 |  |
| 110 | JQ833513 | *Pseudomonas corrugata* | 76 |  |
| 111 | JQ833514 | *Pseudomonas corrugata* | 70 |  |
| 112 | JQ833520 | *Bacillus cereus sensu lato* | 90 |  |
| 113 | JQ833534 | *Pseudomonas lini* | 89 |  |
| 114 | JQ833544 | *Pseudomonas corrugata* | 82 |  |
| 115 | JQ833545 | *Bacillus cereus sensu lato* | 83 | *B*10 |
| 116 | JQ833582 | *Pseudomonas corrugata* | 90 |  |
| 117 | JQ833593 | *Bacillus cereus sensu lato* | 70 |  |
| 118 | JQ833598 | *Pseudomonas corrugata* | 90 |  |
| 119 | JQ833613 | *Pseudomonas lini* | 90 |  |
| 120 | JQ833616 | *Bacillus subtilis* group | 93 |  |
| 121 | JQ834168 | *Bacillus megaterium* | 98 |  |
| 122 |  | N/D | 84 | N11 |
| 123 | JQ834184 | *Bacillus cereus sensu lato* | 80 |  |
| 124 | JQ834187 | *Bacillus subtilis* group | 95 |  |
| 125 | JQ834196 | *Bacillus megaterium* | 91 |  |
| 126 | JQ834198 | *Bacillus subtilis* group | 79 | *B*12 |
| 127 | JQ834212 | *Bacillus cereus sensu lato* | 91 |  |
| 128 | JQ834214 | *Bacillus megaterium* | 81 |  |
| 129 | JQ834233 | *Brevibacillus sp.* | 71 |  |
| 130 | JQ834013 | *Bacillus subtilis* group | 94 |  |
| 131 | JQ834018 | *Bacillus subtilis* group | 86 | *B*13 |
| 132 | JQ834071 | *Bacillus megaterium* | 94 |  |
| 133 | JQ834077 | *Bacillus cereus sensu lato* | 73 |  |
| 134 | N/A | *Bacillus cereus sensu lato* | 63 |  |
| 135 | JQ834270 | *Bacillus subtilis* group | 89 |  |
| 136 | JQ834282 | *Bacillus subtilis* group | 71 |  |
| 137 | JQ834304 | *Bacillus cereus sensu lato* | 81 | *B*14 |
| 138 |  | N/D | 96 |  |
| 139 | JQ834357 | *Bacillus megaterium* | 95 |  |
| 140 | JQ834367 | *Bacillus megaterium* | 66 |  |
| 141 | JQ834358 | *Bacillus subtilis* group | 66 |  |
| 142 | N/A | *Bacillus cereus sensu lato* | 67 |  |
| 143 | JQ834391 | *Bacillus megaterium* | 68 |  |
| 144 | JQ834392 | *Bacillus subtilis* group | 70 |  |
| 145 | JQ834376 | *Bacillus megaterium* | 85 |  |
| 146 | JQ834406 | *Bacillus marisflavi* | 87 |  |
| 147 | JQ834353 | *Bacillus cereus sensu lato* | 74 |  |
| 148 |  | N/D | 94 |  |
| 149 | JQ834461 | *Bacillus subtilis* group | 73 |  |
| 150 | JQ834464 | *Bacillus cereus* *sensu lato* | 93 |  |
| 151 | JQ834465 | *Bacillus cereus sensu lato* | 89 |  |
| 152 | JQ834482 | *Bacillus cereus sensu lato* | 67 |  |
| 153 | JQ834483 | *Bacillus subtilis* group | 73 |  |
| 154 | JQ834502 | *Bacillus cereus sensu lato* | 67 |  |
| 155 | JQ834508 | *Lysinibacillus sp* | 68 |  |
| 156 | JQ834953 | *Bacillus cereus sensu lato* | 67 |  |
| 157 | JQ834955 | *Lysinibacillus sphaericus* | 85 |  |
| 158 | JQ834960 | *Bacillus megaterium* | 79 |  |
| 159 | JQ834970 | *Bacillus subtilis* group | 85 |  |
| 160 | JQ834977 | *Bacillus badius* | 80 |  |
| 161 | JQ834995 | *Bacillus endophyticus* | 70 |  |
| 162 |  | N/D | 73 |  |
| 163 | N/A | *Bacillus cereus sensu lato* | 71 |  |
| 164 | JQ835012 | *Bacillus subtilis* group | 74 |  |
| 165 | JQ835021 | *Lysinibacillus sp.* | 69 |  |
| 166 | JQ835027 | *Bacillus flexus* | 83 |  |
| 167 | JQ835031 | *Bacillus cereus sensu lato* | 80 | *B*15 |
| 168 | JQ835033 | *Bacillus flexus* | 80 |  |
| 169 | JQ835034 | *Bacillus flexus* | 86 |  |
| 170 |  | N/D | 67 |  |
| 171 | N/A | *Lysinibacillus sp.* | 77 |  |
| 172 | JQ835053 | *Bacillus megaterium* | 82 |  |
| 173 | JQ835057 | *Lysinibacillus sphaericus* | 71 |  |
| 174 | JQ835064 | *Bacillus marisflavi* | 85 |  |
| 175 | N/A | *Bacillus fusiformis* | 98 |  |
| 176 | JQ835086 | *Lysinibacillus fusiformis* | 74 |  |
| 177 | JQ835101 | *Lysinibacillus fusiformis* | 75 |  |
| 178 | JQ835102 | *Bacillus megaterium* | 69 |  |
| 179 | N/A | *Bacillus oceanisediminis* | 89 |  |
| 180 | JQ835139 | *Bacillus endophyticus* | 68 |  |
| 181 |  | N/D | 72 |  |
| 182 | JQ835141 | *Bacillus megaterium* | 95 |  |
| 183 | JQ835683 | *Bacillus megaterium* | 74 |  |
| 184 | JQ835677 | *Bacillus flexus* | 91 |  |
| 185 | JQ835690 | *Bacillus flexus* | 80 |  |
| 186 | JQ835695 | *Bacillus flexus* | 80 |  |
| 187 | JQ835705 | *Bacillus cereus sensu lato* | 82 |  |
| 188 |  | N/D | 73 |  |
| 189 | JQ835788 | *Bacillus cereus sensu lato* | 71 |  |
| 190 |  | N/D | 91 |  |
| 191 | JQ835763 | *Bacillus flexus* | 69 |  |
| 192 | JQ835802 | *Bacillus megaterium* | 63 |  |
| 193 |  | N/D | 66 |  |
| 194 |  | N/D | 66 |  |
| 195 |  | N/D | 82 |  |
| 196 |  | N/D | 96 |  |
| 197 |  | N/D | 90 |  |
| 198 | N/A | *Bacillus subtilis* group | 81 | *B*16 |
| 199 |  | N/D | 84 |  |
| 200 |  | N/D | 80 |  |
| 201 |  | N/D | 81 |  |
| 202 |  | N/D | 79 |  |
| 203 |  | N/D | 92 |  |
| 204 |  | N/D | 90 |  |
| 205 |  | N/D | 84 |  |
| 206 |  | N/D | 73 |  |
| 207 |  | N/D | 89 |  |
| 208 |  | N/D | 71 |  |
| 209 | JQ829171 | *Bacillus flexus* | 83 |  |
| 210 | JQ829195 | *Bacillus megaterium* | 79 |  |
| 211 | JQ829196 | *Bacillus flexus* | 68 |  |
| 212 | JQ829207 | *Bacillus aryabhattai* | 77 |  |
| 213 | N/A | *Aquaspirillum itersonii* | 66 |  |
| 214 | JQ830117 | *Bacillus flexus* | 69 |  |
| 215 | JQ830122 | *Bacillus flexus* | 96 |  |
| 216 | JQ830123 | *Bacillus megaterium* | 70 |  |
| 217 | N/A | *Terribacillus sp*. | 70 |  |
| 218 | JQ830179 | *Terribacillus sp*. | 71 |  |
| 219 | JQ830196 | *Bacillus subtilis* group | 80 |  |
| 220 | JQ830209 | *Bacillus flexus* | 71 |  |
| 221 | JQ830210 | *Bacillus megaterium* | 78 |  |
| 222 | JQ830214 | *Bacillus megaterium* | 74 |  |
| 223 | JQ830215 | *Bacillus megaterium* | 90 |  |
| 224 | JQ830223 | *Bacillus megaterium* | 88 |  |
| 225 | JQ830226 | *Paenibacillus borealis* | 80 |  |
| 226 | JQ830227 | *Bacillus niacini* | 86 |  |
| 227 | N/A | *Acinetobacter calcoaceticus* | 70 |  |
| 228 | JQ830236 | *Bacillus megaterium* | 72 |  |
| 229 | JQ830237 | *Bacillus megaterium* | 76 |  |
| 230 | N/A | *Bacillus subtilis* group | 82 | *B*17 |
| 231 | JQ830258 | *Bacillus megaterium* | 79 |  |
| 232 | JQ830259 | *Bacillus megaterium* | 76 |  |
| 233 | JQ830261 | *Bacillus subtilis* group | 81 |  |
| 234 | JQ830268 | *Bacillus megaterium* | 81 |  |
| 235 | JQ830269 | *Bacillus megaterium* | 77 |  |
| 236 | JQ830273 | *Bacillus megaterium* | 70 |  |
| 237 | JQ830277 | *Lysinibacillus fusiformis* | 82 |  |
| 238 | JQ830278 | *Bacillus megaterium* | 88 |  |
| 239 | JQ830279 | *Bacillus megaterium* | 91 |  |
| 240 | JQ830281 | *Bacillus megaterium* | 50 |  |
| 241 | JQ830287 | *Bacillus megaterium* | 72 |  |
| 242 | JQ830299 | *Bacillus cereus sensu lato* | 89 |  |
| 243 | JQ830308 | *Bacillus subtilis* group | 92 |  |
| 244 |  | N/D | 81 |  |
| 245 |  | N/D | 80 |  |
| 246 |  | N/D | 97 |  |
| 247 |  | N/D | 82 |  |
| 248 |  | N/D | 74 |  |
| 249 |  | N/D | 83 |  |
| 250 |  | N/D | 88 |  |
| 251 |  | N/D | 87 |  |
| 252 |  | N/D | 91 |  |
| 253 |  | N/D | 87 |  |
| 254 | N/A | *Bacillus cereus sensu lato* | 85 | *B*18 |
| 255 | N/A | *Bacillus cereus sensu lato* | 71 | *B*19 |
| 256 | JQ830993 | *Bacillus megaterium* | 86 |  |
| 257 | JQ830999 | *Bacillus cereus sensu lato* | 97 |  |
| 258 | JQ831023 | *Bacillus flexus* | 99 |  |
| 259 | JQ831032 | *Bacillus subtilis* group | 90 |  |
| 260 | JQ831035 | *Bacillus endophyticus* | 85 |  |
| 261 | JQ831037 | *Bacillus badius* | 84 |  |
| 262 | JQ831050 | *Bacillus badius* | 84 |  |
| 263 | JQ831055 | *Bacillus megaterium* | 81 |  |
| 264 | JQ831060 | *Bacillus cereus sensu lato* | 85 | *B20* |
| 265 | JQ831062 | *Bacillus megaterium* | 86 |  |
| 266 | JQ831069 | *Bacillus cereus sensu lato* | 91 |  |
| 267 | JQ831071 | *Bacillus megaterium* | 86 |  |
| 268 | JQ831073 | *Bacillus megaterium* | 82 |  |
| 269 |  | N/D | 85 |  |
| 270 |  | N/D | 83 |  |
| 271 |  | N/D | 94 |  |
| 272 |  | N/D | 92 |  |
| 273 |  | N/D | 89 |  |
| 274 |  | N/D | 74 |  |
| 275 |  | N/D | 73 |  |
| 276 |  | N/D | 70 |  |
| 277 |  | N/D | 78 |  |
| 278 |  | N/D | 79 |  |
| 279 |  | N/D | 83 |  |
| 280 | N/A | *Bacillus cereus sensu lato* | 82 | *B*21 |
| 281 | N/A | *Bacillus megaterium* | 74 | *B*22 |
| 282 |  | N/D | 73 |  |
| 283 |  | N/D | 80 |  |
| 284 |  | N/D | 71 |  |
| 285 |  | N/D | 83 |  |
| 286 |  | N/D | 76 |  |
| 287 |  | N/D | 75 |  |
| 288 |  | N/D | 71 |  |
| 289 |  | N/D | 90 |  |
| 290 |  | N/D | 74 |  |
| 291 |  | N/D | 71 |  |
| 292 |  | N/D | 71 |  |
| 293 |  | N/D | 91 |  |
| 294 |  | N/D | 79 |  |
| 295 |  | N/D | 94 |  |
| 296 |  | N/D | 84 |  |
| 297 |  | N/D | 71 |  |
| 298 |  | N/D | 71 |  |
| 299 |  | N/D | 81 |  |
| 300 |  | N/D | 81 |  |
| 301 |  | N/D | 72 |  |
| 302 |  | N/D | 88 |  |
| 303 | JQ832079 | *Bacillus flexus* | 80 |  |
| 304 | JQ832081 | *Bacillus megaterium* | 76 |  |
| 305 | JQ832083 | *Bacillus cereus sensu lato* | 83 |  |
| 306 | JQ832089 | *Bacillus megaterium* | 86 |  |
| 307 | JQ832091 | *Bacillus cereus sensu lato* | 85 |  |
| 308 | JQ832092 | *Bacillus cereus sensu lato* | 88 |  |
| 309 | JQ832097 | *Bacillus megaterium* | 79 |  |
| 310 | JQ832106 | *Bacillus megaterium* | 78 |  |
| 311 | JQ832111 | *Bacillus megaterium* | 77 |  |
| 312 | JQ832118 | *Bacillus megaterium* | 83 |  |
| 313 |  | N/D | 79 |  |
| 314 | JQ832123 | *Bacillus megaterium* | 82 |  |
| 315 | JQ832124 | *Bacillus megaterium* | 83 |  |
| 316 |  | N/D | 80 |  |
| 317 | JQ832141 | *Bacillus flexus* | 78 |  |
| 318 | JQ832142 | *Bacillus flexus* | 76 |  |
| 319 | JQ832143 | *Bacillus megaterium* | 79 |  |
| 320 | JQ832820 | *Bacillus megaterium* | 76 |  |
| 321 | JQ832829 | *Bacillus cereus sensu lato* | 63 |  |
| 322 | JQ832423 | *Bacillus flexus* | 70 |  |
| 323 | JQ832418 | *Bacillus megaterium* | 64 | *B*23 |
| 324 | JQ832488 | *Bacillus subtilis* group | 94 |  |
| 325 | JQ832499 | *Enterobacter cloacae* | 65 |  |
| 326 | JQ832567 | *Lysinibacillus fusiformis* | 62 |  |
| 327 | JQ832569 | *Enterobacter cloacae* | 64 |  |
| 328 |  | N/D | 77 |  |
| 329 |  | N/D | 62 |  |
| 330 | JQ833149 | *Bacillus subtilis* group | 90 |  |
| 331 | JQ833162 | *Bacillus megaterium* | 87 |  |
| 332 | JQ833144 | *Bacillus megaterium* | 81 |  |
| 333 | JQ833201 | *Lysinibacillus fusiformis* | 73 |  |
| 334 | JQ833287 | *Bacillus cereus sensu lato* | 62 |  |
| 335 | JQ833280 | *Bacillus megaterium* | 60 |  |
| 336 | JQ833303 | *Bacillus megaterium* | 65 |  |
| 337 | JQ833707 | *Bacillus flexus* | 68 |  |
| 338 | JQ833672 | *Bacillus megaterium* | 70 |  |
| 339 | JQ833675 | *Bacillus megaterium* | 67 |  |
| 340 | JQ833765 | *Bacillus megaterium* | 61 |  |
| 341 | JQ833789 | *Bacillus flexus* | 75 |  |
| 342 | JQ833756 | *Bacillus cereus sensu lato* | 67 |  |
| 343 | N/A | *Anaerobranca californiensis* | 87 |  |
| 344 | JQ833917 | *Bacillus megaterium* | 82 |  |
| 345 | JQ834598 | *Bacillus subtilis* group | 72 |  |
| 346 | JQ834607 | *Bacillus megaterium* | 89 |  |
| 347 | JQ834649 | *Pseudomonas stutzeri* | 85 |  |
| 348 | JQ834672 | *Bacillus cereus sensu lato* | 86 |  |
| 349 | JQ834691 | *Bacillus megaterium* | 79 |  |
| 350 | JQ834737 | *Bacillus megaterium* | 77 |  |
| 351 | JQ835192 | *Bacillus cereus sensu lato* | 70 |  |
| 352 | JQ835234 | *Bacillus subtilis* group | 73 |  |
| 353 | JQ835299 | *Bacillus subtilis* group | 74 |  |
| 354 | JQ835289 | *Bacillus megaterium* | 78 |  |
| 355 | JQ835291 | *Bacillus megaterium* | 76 |  |
| 356 | JQ835314 | *Bacillus aryabhattai* | 74 |  |
| 357 | JQ835336 | *Bacillus megaterium* | 73 |  |
| 358 | JQ835385 | *Bacillus megaterium* | 82 |  |
| 359 | JQ835396 | *Bacillus megaterium* | 74 |  |
| 360 | JQ835403 | *Lysinibacillus fusiformis* | 81 |  |
| 361 | JQ835408 | *Bacillus cereus* *sensu lato* | 75 |  |
| 362 | JQ835418 | *Bacillus megaterium* | 89 |  |
| 363 | JQ835429 | *Bacillus megaterium* | 80 |  |
| 364 | JQ835429 | *Bacillus cereus sensu lato* | 72 |  |
| 365 | JQ835834 | *Bacillus cereus sensu lato* | 72 |  |
| 366 | JQ835838 | *Bacillus cereus sensu lato* | 73 |  |
| 367 | JQ835859 | *Bacillus cereus* *sensu lato* | 72 | *B*24 |
| 368 | JQ835870 | *Bacillus cereus* *sensu lato* | 72 |  |
| 369 | JQ835881 | *Lysinibacillus fusiformis* | 72 |  |
| 370 | JQ835905 | *Lysinibacillus fusiformis* | 76 |  |
| 371 | JQ835875 | *Pseudomonas chlororaphis* | 76 |  |
| 372 | JQ835919 | *Bacillus flexus* | 92 |  |
| 373 | JQ835946 | *Bacillus cereus sensu lato* | 93 | *B*25 |
| 374 | JQ835949 | *Bacillus cereus sensu lato* | 86 |  |
| 375 | JQ835950 | *Bacillus cereus sensu lato* | 89 |  |
| 376 | JQ835987 | *Bacillus cereus sensu lato* | 92 |  |
| 377 | JQ835998 | *Bacillus cereus sensu lato* | 91 |  |
| 378 |  | N/D | 88 |  |
| 379 |  | N/D | 87 |  |
| 380 |  | N/D | 90 |  |
| 381 | JQ829267 | *Enterobacter asburiae* | 90 |  |
| 382 | JQ829276 | *Bacillus subtilis* group | 88 |  |
| 383 | JQ829288 | *Enterobacter hormaechei* | 91 |  |
| 384 | JQ829294 | *Enterobacter cloacae* | 89 |  |
| 385 | JQ829301 | *Enterobacter hormaechei* | 91 |  |
| 386 | JQ829310 | *Enterobacter cloacae* | 90 |  |
| 387 | JQ829353 | *Enterobacter cloacae* | 93 |  |
| 388 | JQ829362 | *Enterobacter hormaechei* | 92 |  |
| 389 | JQ829425 | *Enterobacter cloacae* | 75 |  |
| 390 | JQ829426 | *Enterobacter asburiae* | 93 |  |
| 391 | JQ829479 | *Enterobacter asburiae* | 83 |  |
| 392 | JQ829460 | *Enterobacter hormaechei* | 84 |  |
| 393 | JQ829461 | *Enterobacter hormaechei* | 87 |  |
| 394 | JQ829518 | *Enterobacter hormaechei* | 82 |  |
| 395 | JQ830336 | *Bacillus cereus sensu lato* | 82 |  |
| 396 | JQ830343 | *Enterobacter hormaechei* | 84 |  |
| 397 | JQ830356 | *Enterobacter cancerogenus* | 82 |  |
| 398 | JQ830403 | *Bacillus arbutinivorans* | 85 |  |
| 399 | JQ830426 | *Enterobacter hormaechei* | 79 |  |
| 400 | JQ830443 | *Enterobacter asburiae* | 86 |  |
| 401 | JQ830486 | *Enterobacter asburiae* | 87 |  |
| 402 | JQ830498 | *Enterobacter aerogenes* | 81 |  |
| 403 | JQ830534 | *Enterobacter hormaechei* | 75 |  |
| 404 | JQ831079 | *Stenotrophomonas maltophilia* | 71 |  |
| 405 | JQ831086 | *Bacillus oceanisediminis* | 72 |  |
| 406 | JQ831089 | *Bacillus subtilis* group | 75 |  |
| 407 |  | N/D | 83 |  |
| 408 | JQ831141 | *Arthrobacter globiformis* | 74 |  |
| 409 | JQ831159 | *Agrobacterium tumefaciens* | 75 |  |
| 410 | JQ831175 | *Bacillus subtilis* group | 77 |  |
| 411 | JQ831176 | *Bacillus subtilis* group | 87 |  |
| 412 | JQ831182 | *Pseudomonas pseudoalcaligenes* | 78 |  |
| 413 | N/A | *Bacillus subtilis* group | 85 |  |
| 414 | N/A | Uncultured bacterium clone | 87 |  |
| 415 | JQ831198 | *Bacillus altitudinis* | 90 |  |
| 416 | JQ831199 | *Agrobacterium rubi* | 90 |  |
| 417 | JQ831210 | *Bacillus subtilis* group | 79 |  |
| 418 | JQ831216 | *Bacillus cereus sensu lato* | 82 |  |
| 419 | N/A | *Brevibacillus brevis* | 79 |  |
| 420 | N/A | *Bacillus drentensis* | 81 |  |
| 421 | JQ831242 | *Stenotrophomonas maltophilia* | 83 |  |
| 422 | JQ831248 | *Bacillus cereus sensu lato* | 74 | *B*26 |
| 423 | JQ831249 | *Bacillus cereus sensu lato* | 77 |  |
| 424 | N/A | *Stenotrophomonas maltophilia* | 92 |  |
| 425 |  | N/D | 75 | N27 |
| 426 | JQ831276 | *Paenibacillus xylanilyticus* | 82 |  |
| 427 | JQ831280 | *Bacillus megaterium* | 92 |  |
| 428 | JQ831284 | *Stenotrophomonas maltophilia* | 79 |  |
| 429 | JQ831294 | *Agrobacterium tumefaciens* | 88 |  |
| 430 | N/A | *Bacillus* sp. | 76 | *B*35 |
| 431 |  | N/D | 77 |  |
| 432 |  | N/D | 74 |  |
| 433 |  | N/D | 75 |  |
| 434 |  | N/D | 73 |  |
| 435 |  | N/D | 92 |  |
| 436 | N/A | *Bacillus subtilis* group | 82 |  |
| 437 | JQ831833 | *Bacillus cereus sensu lato* | 94 |  |
| 438 |  | N/D | 86 |  |
| 439 | JQ831889 | *Bacillus megaterium* | 76 |  |
| 440 |  | N/D | 84 |  |
| 441 | JQ831907 | *Arthrobacter globiformis* | 92 |  |
| 442 | N/A | *Bacillus cereus sensu lato* | 75 |  |
| 443 | JQ831917 | *Bacillus flexus* | 82 |  |
| 444 | N/A | *Bacillus cereus sensu lato* | 89 |  |
| 445 |  | N/D | 94 |  |
| 446 | JQ832587 | *Bacillus cereus* *sensu lato* | 79 | *B*28 |
| 447 |  | N/D | 89 |  |
| 448 | JQ832589 | *Bacillus cereus sensu lato* | 77 |  |
| 449 |  | N/D | 76 |  |
| 450 | JQ832600 | *Bacillus oceanisediminis* | 83 |  |
| 451 | JQ832601 | *Bacillus cereus sensu lato* | 72 | *B*29 |
| 452 |  | N/D | 77 |  |
| 453 |  | N/D | 80 |  |
| 454 | JQ832624 | *Bacillus oceanisediminis* | 76 |  |
| 455 | N/A | *Bacillus cereus sensu lato* | 89 |  |
| 456 | JQ832645 | *Bacillus cereus sensu lato* | 92 |  |
| 457 | JQ832668 | *Bacillus cereus* *sensu lato* | 83 | *B*30 |
| 458 | JQ832678 | *Bacillus cereus* *sensu lato* | 75 |  |
| 459 | JQ832688 | *Bacillus cereus* *sensu lato* | 79 |  |
| 460 | JQ832702 | *Bacillus cereus* *sensu lato* | 75 | *B*31 |
| 461 |  | N/D | 80 |  |
| 462 | N/A | *Bacillus cereus sensu lato* | 90 | *B*32 |
| 463 |  | N/D | 81 |  |
| 464 |  | N/D | 79 |  |
| 465 |  | N/D | 80 |  |
| 466 |  | N/D | 85 |  |
| 467 |  | N/D | 80 |  |
| 468 |  | N/D | 85 |  |
| 469 |  | N/D | 85 |  |
| 470 |  | N/D | 85 |  |
| 471 |  | N/D | 87 |  |
| 472 |  | N/D | 86 |  |
| 473 |  | N/D | 87 |  |
| 474 | N/A | *Bacillus cereus sensu lato* | 86 | *B*34 |
| 475 |  | N/D | 93 |  |
| 476 |  | N/D | 79 |  |
| 477 |  | N/D | 90 |  |
| 478 |  | N/D | 84 |  |
| 479 | JQ833348 | *Arthrobacter globiformis* | 85 |  |
| 480 | JQ833929 | *Bacillus cereus sensu lato* | 76 | *B*33 |
| 481 | N/A | *Bacillus subtilis* group | 86 |  |
| 482 | JQ833955 | *Bacillus cereus sensu lato* | 78 |  |
| 483 | JQ833957 | *Bacillus cereus sensu lato* | 74 |  |
| 484 | N/A | *Geobacillus thermodenitrificans* | 78 |  |
| 485 | N/A | *Bacillus cereus sensu lato* | 78 |  |
| 486 |  | N/D | 82 |  |
| 487 | JQ833976 | *Bacillus bataviensis* | 77 |  |
| 488 | JQ833988 | *Bacillus cereus sensu lato* | 77 |  |
| 489 |  | N/D | 79 |  |
| 490 |  | N/D | 81 |  |
| 491 |  | N/D | 79 |  |
| 492 |  | N/D | 78 |  |
| 493 | N/A | *Bacillus cereus sensu lato* | 85 | *B*36 |
| 494 |  | N/D | 75 |  |
| 495 |  | N/D | 80 | N37 |
| 496 |  | N/D | 78 |  |
| 497 |  | N/D | 76 |  |
| 498 |  | N/D | 78 |  |
| 499 | N/A | *Bacillus cereus sensu lato* | 81 | *B*38 |
| 500 |  | N/D | 77 |  |
| 501 |  | N/D | 75 |  |
| 502 |  | N/D | 75 |  |
| 503 |  | N/D | 84 |  |
| 504 |  | N/D | 76 |  |
| 505 |  | N/D | 83 |  |
| 506 |  | N/D | 87 |  |
| 507 |  | N/D | 91 |  |
| 508 |  | N/D | 84 |  |
| 509 | JQ834802 | *Bacillus cereus sensu lato* | 86 |  |
| 510 | JQ834819 | *Bacillus cereus sensu lato* | 81 |  |
| 511 | JQ834825 | *Bacillus cereus sensu lato* | 77 |  |
| 512 | JQ834832 | *Bacillus cereus sensu lato* | 81 |  |
| 513 | JQ834842 | *Bacillus cereus sensu lato* | 95 |  |
| 514 | N/A | Uncultured bacterium clone | 83 |  |
| 515 | N/A | *Bacillus cereus sensu lato* | 79 |  |
| 516 |  | N/D | 78 |  |
| 517 | JQ834824 | *Bacillus firmus* | 76 |  |
| 518 |  | N/D | 85 |  |
| 519 |  | N/D | 77 |  |
| 520 | N/A | *Bacillus cereus sensu lato* | 91 |  |
| 521 | JQ835485 | *Bacillus cereus sensu lato* | 79 |  |
| 522 | JQ835547 | *Bacillus subtilis* group | 78 |  |
| 523 | JQ835589 | *Bacillus cereus* *sensu lato* | 76 |  |
| 524 | JQ835602 | *Bacillus cereus sensu lato* | 84 |  |
| 525 | JQ835641 | *Pseudomonas putida* | 77 |  |
| 526 |  | N/D | 86 | N39 |
| 527 |  | N/D | 96 |  |
| 528 | N/A | *Bacillus asahii* | 77 |  |
| 529 | N/A | *Acinetobacter rhizosphaerae* | 81 |  |
| 530 | JQ836137 | *Acinetobacter calcoaceticus* | 89 |  |
| 531 |  | N/D | 88 |  |
| 532 | N/A | *Bacillus cereus* *sensu lato* | 90 |  |
| 533 | JQ836158 | *Bacillus cereus sensu lato* | 80 |  |
| 534 | N/A | *Bacillus cereus* *sensu lato* | 84 |  |
| 535 | JQ836159 | *Bacillus sonorensis* | 85 |  |
| 536 | N/A | *Bacillus cereus* *sensu lato* | 85 |  |
| 537 | N/A | *Bacillus cereus sensu lato* | 94 |  |
| 538 |  | N/D | 87 |  |
| 539 | N/A | *Bacillus subtilis* group | 91 |  |
| 540 | JQ836172 | *Bacillus cereus sensu lato* | 81 |  |
| 541 | JQ836173 | *Bacillus cereus sensu lato* | 84 |  |
| 542 |  | N/D | 95 | N40 |
| 543 | N/A | *Bacillus cereus sensu lato* | 89 | *B*41 |
| 544 | JQ836198 | *Bacillus cereus sensu lato* | 89 |  |
| 545 | JQ829546 | *Pantoea dispersa* | 78 |  |
| 546 | JQ829551 | *Pantoea dispersa* | 83 |  |
| 547 | JQ829555 | *Bacillus subtilis* group | 89 |  |
| 548 | JQ829577 | *Pantoea dispersa* | 78 |  |
| 549 | JQ829580 | *Bacillus cereus* *sensu lato* | 98 |  |
| 550 | JQ829606 | *Bacillus megaterium* | 80 |  |
| 551 | JQ829658 | *Bacillus megaterium* | 87 |  |
| 552 | JQ829661 | *Bacillus flexus* | 86 |  |
| 553 | JQ829684 | *Bacillus megaterium* | 93 |  |
| 554 | JQ829694 | *Bacillus megaterium* | 87 |  |
| 555 | JQ829752 | *Bacillus cereus* *sensu lato* | 79 |  |
| 556 |  | N/D | 82 |  |
| 557 | JQ829755 | *Bacillus megaterium* | 76 |  |
| 558 | JQ829723 | *Bacillus cereus* *sensu lato* | 90 |  |
| 559 |  | N/D | 96 |  |
| 560 |  | N/D | 88 |  |
| 561 |  | N/D | 85 |  |
| 562 |  | N/D | 91 |  |
| 563 |  | N/D | 88 |  |
| 564 |  | N/D | 91 |  |
| 565 |  | N/D | 86 |  |
| 566 |  | N/D | 92 |  |
| 567 |  | N/D | 94 |  |
| 568 |  | N/D | 89 |  |
| 569 |  | N/D | 89 |  |
| 570 |  | N/D | 90 |  |
| 571 |  | N/D | 92 |  |
| 572 |  | N/D | 90 |  |
| 573 | JQ830598 | *Enterobacter cloacae* | 89 |  |
| 574 | JQ830601 | *Enterobacter cloacae* | 87 |  |
| 575 | JQ830608 | *Enterobacter cloacae* | 91 |  |
| 576 | JQ830629 | *Enterobacter cloacae* | 84 |  |
| 577 | N/A | *Bacillus subtilis* group | 85 |  |
| 578 | JQ830649 | *Enterobacter cloacae* | 86 |  |
| 579 | JQ830665 | *Enterobacter cloacae* | 88 |  |
| 580 | JQ830666 | *Enterobacter cloacae* | 89 |  |
| 581 | JQ830689 | *Enterobacter sp.* | 89 |  |
| 582 | JQ830696 | *Klebsiella pneumonae* | 89 |  |
| 583 | JQ830697 | *Enterobacter cloacae* | 88 |  |
| 584 |  | N/D | 92 |  |
| 585 | JQ830734 | *Bacillus subtilis* group | 88 |  |
| 586 | JQ831337 | *Bacillus megaterium* | 88 |  |
| 587 | N/A | *Bacillus cereus sensu lato* | 87 |  |
| 588 |  | N/D | 90 |  |
| 589 | JQ831449 | *Enterobacter ludwigii* | 90 |  |
| 590 |  | N/D | 75 |  |
| 591 |  | N/D | 76 |  |
| 592 |  | N/D | 74 |  |
| 593 |  | N/D | 88 |  |
| 594 |  | N/D | 74 |  |
| 595 |  | N/D | 59 |  |
| 596 |  | N/D | 89 |  |
| 597 |  | N/D | 93 |  |
| 598 |  | N/D | 93 |  |
| 599 | N/A | *Bacillus cereus sensu lato* | 66 |  |
| 600 |  | N/D | 69 |  |
| 601 |  | N/D | 75 |  |
| 602 |  | N/D | 79 |  |
| 603 | JQ831992 | *Bacillus cereus* *sensu lato* | 70 |  |
| 604 | JQ832028 | *Bacillus megaterium* | 72 |  |
| 605 | JQ832030 | *Bacillus subtilis* group | 79 |  |
| 606 | JQ832046 | *Bacillus megaterium* | 60 |  |
| 607 |  | N/D | 60 |  |
| 608 |  | N/D | 70 |  |
| 609 |  | N/D | 60 |  |
| 610 |  | N/D | 85 |  |
| 611 |  | N/D | 95 |  |
| 612 |  | N/D | 70 |  |
| 613 |  | N/D | 63 |  |
| 614 |  | N/D | 72 |  |
| 615 |  | N/D | 81 |  |
| 616 |  | N/D | 60 |  |
| 617 | JQ832716 | *Pseudomonas fluorescens* | 70 |  |
| 618 | JQ832727 | *Pseudomonas putida* | 60 |  |
| 619 | N/A | *Pseudomonas fluorescens* | 74 | *Ps*42 |
| 620 |  | N/D | 60 |  |
| 621 | JQ832755 | *Pseudomonas fluorescens* | 81 |  |
| 622 | JQ832768 | *Pseudomonas putida* | 76 |  |

^1^Letters preceding the isolate number indicate the genus of that particular isolate. *B* refers to *Bacillus*, N is not determined, *Ps* is *Pseudomonas*, *Pa* is *Paenibacillus* and U is uncultured bacterium.

^2^N/D stands for not determined.

^3^N/A stands for not assigned.
